# ScRNA‐Seq Reveals Jiangfu Zhixie Formula Alleviates IBS‐D by Inhibiting Macrophage M1 Polarization through HIF‐1α‐Glycolysis Axis

**DOI:** 10.1002/iid3.70599

**Published:** 2026-11-05

**Authors:** Xiaosong Xu, Yao Du, Lipeng Du, Wanning Zhang, Bowen Guo, Yanru Cai, Tianyi Zhang

**Affiliations:** ^1^ Research Center, Hebei Provincial Hospital of Chinese Medicine Hebei China; ^2^ Department of Spleen and Stomach Diseases Hebei Provincial Hospital of Chinese Medicine Hebei China

**Keywords:** glycolysis, HIF‐1 α, irritable bowel syndrome with diarrhea, Jiangfu Zhixie formula, macrophage polarization, single‐cell rna sequencing

## Abstract

**Background:**

Irritable bowel syndrome with diarrhea (IBS‐D) remains a challenging condition to manage due to its chronic nature and the limited long‐term efficacy of current treatments. The traditional Chinese herbal formula Jiangfu Zhixie (JFZX) has shown clinical benefits, but its underlying mechanisms remain unclear.

**Aim:**

This study aimed to investigate the therapeutic effects of JFZX on IBS‐D and elucidate its molecular mechanisms, focusing on macrophage polarization and the HIF‐1α‐glycolysis axis.

**Methods:**

An IBS‐D mouse model was established by senna leaf infusion combined with restraint stress. Mice were treated with varying doses of JFZX or pinaverium bromide (positive control) for 14 days. Diarrhea symptoms, visceral hypersensitivity, and intestinal barrier integrity were assessed. Single‐cell RNA sequencing (scRNA‐seq) was performed on colon tissues to analyze cellular composition and intercellular interactions. In vitro, LPS‐stimulated RAW264.7 macrophages were treated with JFZX‐medicated serum to evaluate M1 polarization, cytokine secretion (IL‐1β, TNF‐α), and expression of HIF‐1α and glycolysis‐related proteins (GLUT1, LDHA).

**Results:**

JFZX treatment significantly ameliorated diarrhea, reduced visceral hypersensitivity, and restored intestinal barrier integrity in IBS‐D mice, accompanied by upregulation of tight junction proteins ZO‐1 and Occludin. scRNA‐seq revealed that JFZX reshaped the colonic cellular landscape, attenuating myeloid‐epithelial interactions and reducing the proportion of pro‐inflammatory M1‐like macrophages. Mechanistically, HIF‐1α was identified as a key driver of M1 polarization in IBS‐D. JFZX suppressed HIF‐1α expression and its downstream glycolytic pathway (GLUT1, LDHA) both in vivo and in vitro, thereby inhibiting M1 macrophage polarization and decreasing IL‐1β and TNF‐α secretion in LPS‐stimulated macrophages.

**Conclusion:**

JFZX alleviates IBS‐D by inhibiting M1 macrophage polarization through suppression of the HIF‐1α‐glycolysis axis, highlighting its potential as a therapeutic agent for IBS‐D and identifying HIF‐1α as a promising target for intervention.

## 1 Introduction

Irritable bowel syndrome (IBS) is a chronic functional bowel disease, which is estimated to affect 4% to 10% of the world [1]. Patients experience recurrent abdominal pain or discomfort linked to changes in stool frequency or consistency. Clinically, cases are classified by major faecal modes: constipation type (IBS‐C), diarrhea (IBS‐D) and mixed type (IBS‐M). IBS D is the most common subtype and accounts for about 54% of cases [2]. Current management centers on symptom control with antispasmodics, antidiarrheals, and probiotics. However, the chronic, relapsing course of IBS‐D and the modest long‐term efficacy of existing drugs underscore the need for better options [3]. Against this backdrop, traditional Chinese herbal medicines have drawn growing interest. Their multi‐component, multi‐target nature and generally favorable safety profile position them as a promising avenue for IBS‐D treatment.

Jiangfu Zhixie Formula (JFZX) is an herbal preparation derived from two classic formulas in Zhang Zhongjing's *Treatise on Cold Damage and Miscellaneous Diseases*: Zhenwu Tang and Xiaoqinglong Tang. Zhenwu Tang is traditionally indicated for warming yang and promoting diuresis in patterns of spleen–kidney yang deficiency with internal water–dampness. Xiaoqinglong Tang, by contrast, addresses external cold with internal fluid retention by releasing the exterior, dispersing cold, and warming the lungs to resolve phlegm–fluid. JFZX integrates the core principles of both formulas. It is designed to warm and tonify the spleen and kidney, disperse lung cold, promote diuresis, and regulate qi movement—features that suit complex presentations involving spleen–kidney yang deficiency accompanied by cold–dampness obstruction and qi dysregulation. The formula contains 14 herbal components (Table S1). Preliminary clinical reports suggest that JFZX can alleviate abdominal pain and diarrhea in IBS‐D patients [4]. Rodent studies also indicate it may help repair the intestinal mucosal barrier [5, 6]. Nevertheless, the specific molecular mechanisms and pharmacological targets through which JFZX acts remain unclear. Elucidating these mechanisms is essential both to support its clinical application and to uncover new therapeutic strategies for IBS–D.

The pathophysiology of IBS is now understood to involve multiple interconnected factors, including immune dysregulation, gut microbiota imbalance, altered intestinal motility, visceral hypersensitivity, and compromised barrier function [7, 8]. A key event in this process is the breakdown of intestinal barrier integrity. Increased permeability allows luminal antigens and microbial products to translocate, which then activates the mucosal immune system—a mechanism thought to drive the chronicity of IBS symptoms^[1]^. Within this setting, intestinal macrophages are crucial for maintaining homeostasis. In the face of chronic inflammation, these cells frequently shift toward a pro‐inflammatory, M1‐like phenotype [9, 10]. This polarization is closely tied to metabolic reprogramming, orchestrated largely by hypoxia‐inducible factor‐1α (HIF‐1α). HIF‐1α acts as a master regulator, enabling cellular adaptation to both hypoxic and inflammatory conditions [11]. In the chronically inflamed intestinal microenvironment of IBS, local tissue hypoxia coexists with inflammatory signals—such as NF‐κB activation—that further stabilize HIF‐1α protein [12]. Once active, HIF‐1α transcriptionally upregulates key glycolytic enzymes such as GLUT1, HK2, and PKM2, pushing macrophages toward a glycolytic metabolic state [13]. Therefore, the HIF‐1α–glycolysis axis emerges as a critical connector—linking the disease's inflammatory microenvironment to metabolic rewiring and, ultimately, to the pro‐inflammatory polarization of macrophages in IBS.

Single cell RNA sequencing (scRNA‐seq) can query transcriptional heterogeneity, cell status, and lineage locus in complex tissues with high resolution without deviation [14]. Based on this capability, we hypothesized that the therapeutic effect of JFZX in IBS‐D involves modulation of the HIF‐1α pathway within intestinal macrophages. We propose that JFZX acts by inhibiting HIF‐1α activation, which in turn dampens glycolysis, reduces M1 macrophage polarization, alleviates intestinal inflammation, and promotes barrier repair. To test this hypothesis, we used scRNA‐seq to define the cellular and transcriptional landscape of the colon in IBS‐D and to assess how JFZX reshapes it. Our study aims to reveal new mechanistic insights into JFZX's action and to identify candidate targets for future IBS‐D therapy.

## 2 Materials and Methods

### 2.1 Preparation of JFZX and Senna Infusion Solutions

All crude herbs were sourced from the Hebei Provincial Hospital of Chinese Medicine (Shijiazhuang, China). We verified the botanical identity of each component against the Medicinal Plant Names Services (MPNS, http://mpns.kew.org). The full formulation of JFZX is listed in Table S1. To prepare the JFZX decoction, the herb was immersed in eight volumes of distilled water for 30 min, followed by boiling and filtration for 1 h. Subsequently, the residue was subjected to a second boiling with six volumes of water for an additional hour. The resultant solution was concentrated twice to achieve a final concentration of 1.1 g/mL.

The senna leaf infusion was made by steeping weighed leaves in 8 times of boiling water for 25 min, followed by filtration. This step was repeated once, and the combined filtrates were concentrated to 0.25 g/mL.

Mouse doses were calculated based on body surface area, using a conversion factor of 12 times the human clinical dose [15]. We set a medium dose of 11.34 g/kg/day, and from this defined a low dose (half the medium, 5.67 g/kg/day) and a high dose (twice the medium, 22.68 g/kg/day). All treatments were administered by oral gavage at a volume of 20 mL/kg.

### 2.2 Animal Experimental Protocol

Sixty male C57BL/6 mice (aged 8 weeks, weighing 18–20 g, SPF grade) were procured from Beijing HFK Bioscience Co. Ltd. (SCXK (Jing) 2024‐0003) and maintained under specific pathogen‐free conditions, with a 12‐h light/dark cycle and controlled temperature and humidity. The animal experimental protocol received approval from the Animal Ethics Committee of Hebei Provincial Hospital of Traditional Chinese Medicine (IACUC‐HPHCM‐2024105).

Following a 1‐week acclimation period, mice were randomly divided into six groups (*n* = 10/group): Control, Model, JFZX low‐dose (JFZX‐L), JFZX medium‐dose (JFZX‐M), JFZX high‐dose (JFZX‐H), and positive control (PC). The IBS‐D model was established following a published protocol [3, 16]. Briefly, mice received daily oral gavage of senna leaf infusion (5 g/kg/day) for 14 days. 1 h after each gavage, they were subjected to restraint stress for 1 h/day throughout the same period.

After model establishment, Control and Model groups received sterile water. The JFZX‐L, JFZX‐M, and JFZX‐H groups were treated with JFZX at 5.67,11.34, and 22.68 g/kg/day, respectively. The PC group received Pinaverium Bromide (19 mg/kg/day) [17]. Treatments were given daily for 14 consecutive days.

### 2.3 Diarrhea Rate and Fecal Water Content

Following the treatment, we documented the incidence of diarrhea among the mice in each group. The diarrhea rate was calculated as (number of animals with diarrhea/total animals in group) × 100%. Fecal consistency was scored using the Bristol Stool Form Scale [18].

### 2.4 Visceral Sensitivity Assessment

Visceral sensitivity was evaluated using the abdominal withdrawal reflex (AWR) scoring system [3, 19]. The mice underwent a 12‐h fasting period with ad libitum access to water, followed by anesthesia induced via intraperitoneal injection of 0.3% sodium pentobarbital at a dosage of 1 mL per 100 g of body weight. A lubricated 6 F balloon‐tipped catheter was inserted approximately 1 cm into the rectum, secured at the base of the tail, and connected to a 1 mL syringe. After 15 min of post‐anesthetic recovery in a clear enclosure, the balloon was inflated gradually with air. The minimum inflation volume that triggered a distinct abdominal lift with dorsal muscle contraction was recorded as the visceral pain threshold (Table S2).

### 2.5 Sample Collection and Processing

Prior to tissue harvesting, the mice were subjected to an overnight fast while being allowed unrestricted access to water. Subsequently, they were anesthetized via an intraperitoneal injection of 0.3% sodium pentobarbital at a dosage of 1 mL/100 g. A midline laparotomy was performed to excise the colon, and the harvested tissues were immediately rinsed in ice‐cold 0.9% saline solution to eliminate any residual fecal matter, adjacent adipose tissue, and mesenteric attachments.

### 2.6 scRNA‐Seq and Data Analysis

Colon samples were subjected to scRNA‐seq on the 10× Genomics platform (Beijing SeekGene BioSciences Co. Ltd., China). Raw reads were processed with Cell Ranger (version 8.0) for demultiplexing, barcode correction, and alignment against the GRCh38/GRCm38 combined reference. Subsequent analysis was performed in Seurat (version 4.1.1). We normalized the data, performed dimensionality reduction via PCA, and visualized cells in two dimensions using UMAP. Cells were filtered by requiring 500–5,000 detected genes and > 500 UMIs; cells where > 20% of reads mapped to mitochondrial genes were excluded. Clusters were defined based on shared transcriptional profiles, and marker genes for each cluster were identified with the FindAllMarkers function. Additional analyses—Kyoto Encyclopedia of Genes and Genomes (KEGG) enrichment, cell‐cell communication inference, and pseudotime trajectory construction—were conducted using the SeekSoul Online platform (https://seeksoul.online).

### 2.7 Hematoxylin‐Eosin (H&E) Staining

Colon tissues embedded in paraffin were sliced into 5 µm sections, deparaffinized in xylene, and then rehydrated through a descending series of ethanol. Following a brief rinse with distilled water, the specimens were stained with hematoxylin for a duration of 4 min, differentiated in 1% acid alcohol solution, blued in 0.7% ammonia water solution, and subsequently counterstained with eosin for 5 min. Ultimately, the sections underwent dehydration through a graded series of ethanol concentrations, were cleared in xylene, and mounted using neutral balsam. Morphological examination was conducted utilizing a light microscope (ZEISS, Germany), following an established protocol^[20]^.

### 2.8 Preparation of Drug‐Containing Serum

Serum containing JFZX was obtained from rats following an established protocol [20]. After 1 week of acclimatization, rats were assigned to either a control group or a JFZX treatment group. Animals in the treatment group received JFZX orally (11.34 g/kg/day) for three consecutive days, at a dose equivalent to 6 times the human clinical dose based on body surface area [15]. Blood was collected from the abdominal aorta under anesthesia 1 h after the final administration. Serum was separated by centrifugation and stored at –80°C until use.

### 2.9 Cell Culture

RAW264.7 macrophages (Biocompete Inc.,Zhenjiang, China) were cultured in Dulbecco's Modified Eagle Medium (DMEM) supplemented with 10% fetal bovine serum (FBS) at 37°C under 5% CO_2_. Cells were passaged at ~80% confluence, counted, and seeded into 6‐well plates at 2 × 10^5^ cells/mL for experiments. RAW264.7 cells were stimulated with LPS (L2880, Sigma Aldrich) at a concentration of 100 ng/mL for 24 h, inducing polarization of RAW264.7 cells towards M1 macrophage phenotype [21].

### 2.10 CCK‐8 Cell Viability Assay

RAW 264.7 cells were seeded into 96‐well plates at a density of 8 × 10^3^ cells per well. Following this, the cells were treated with JFZX‐medicated serum at concentrations of 0%, 2.5%, 5.0%, 10.0%, 20.0%, 40.0%, and 60%, with three wells per concentration serving as replicates. After 24 h and 48 h of incubation, the culture medium was replaced with 100 μL of DMEM that included 10 μL of CCK‐8 reagent (BMU106‐CN, Abbkine Scientific Co. Ltd., Hubei, China), in accordance with the manufacturer's instructions. The cells were then incubated for an extra 2 h at 37°C in a humidified environment with 5% CO_2_. Absorbance readings were taken at 450 nm using a microplate reader (Spectro Star Nano, Germany). The cell viability was determined as the percentage of absorbance compared to that of the untreated control group. The experimental procedure was adapted from Liu et al. [22]. The effects of the indicated concentrations of JFZX‑medicated serum on the viability of LPS‑stimulated RAW 264.7 cells were assessed at 0, 24, 48, and 72 h.

### 2.11 Immunofluorescence (IF) Staining

Raw264.7 cells were cultured in 6‐well plates featuring glass coverslips. Following stimulation with LPS, the cells underwent fixation using 4% paraformaldehyde (P1110, Solarbio, Beijing, China), were permeabilized with 0.1% Triton X‐100 (T8200, Solarbio, Beijing, China), and were subsequently blocked with 5% normal goat serum (ab150167, Abcam, England) for 1 h at 37°C. The cells were exposed to primary antibodies (1:100) overnight at 4°C, and then incubated with the corresponding fluorescent secondary antibodies (1:100) for 90 min at 37°C in the absence of light. Nuclei were stained with DAPI (S2110, Solarbio, Beijing, China). Images were obtained using a fluorescence microscope (Nikon, Japan) and were analyzed utilizing Image Pro Plus 6.0 software [23].

#### 2.11.1 Enzyme‐Linked Immunosorbent Assay (ELISA)

Cell culture supernatants were subjected to centrifugation at a speed of 12,000 rpm for a duration of 10 min, with a constant temperature maintained at 4 degrees Celsius. Following this process, we assessed the concentrations of Interleukin 1 beta (IL‐1β) and Tumor Necrosis Factor alpha (TNF‐α) in the supernatants. The concentrations of these cytokines were determined using commercially available ELISA kits, specifically IL‐1β (J24057) and TNF‐α (J24029), both sourced from JILID Biotechnology located in Wuhan, China. In order to ensure the reliability of the results, each sample was analyzed in triplicate, and the absorbance readings were taken at a wavelength of 450 nanometers.

#### 2.11.2 Western Blot (WB) Analysis

Cells and tissues were lysed using RIPA buffer (R002, Solarbio, Beijing, China), and the protein extracts were separated by 10% SDS‐PAGE (AR0138, BOSTER Biological Technology, Wuhan, China), followed by electrotransfer to PVDF membranes. The membranes underwent a blocking step for 2 h at room temperature and were subsequently incubated overnight at 4°C with a primary antibody (1:800). After thorough washing with TBST (AR0195‐10, BOSTER Biological Technology, Wuhan, China), the blots were treated with an HRP‐conjugated secondary antibody. The protein bands were detected using the SuperKine™ Ultra‐sensitive ECL Chemiluminescence Detection Kit (BMU102‐CN, Abbkine, Wuhan, China) and analyzed densitometrically with Image Pro Plus 6.0. Western blotting was performed as described [23].

#### 2.11.3 Statistical Analysis

Statistical evaluations were conducted utilizing GraphPad Prism (version 8.0). All quantitative information is presented as mean ± SD. The comparisons across several groups were analyzed using one‐way ANOVA with Dunnett's post hoc test applied subsequently. A *p*‐value of less than 0.05 was regarded as statistically significant.

## 3 Results

### 3.1 JFZX Ameliorated Diarrheal Symptoms in IBS‐D Mice

To evaluate the therapeutic effect of JFZX on IBS‐D, we established a murine model of IBS‐D (Figure 1A). Compared with the control group, model mice showed significantly reduced body weight gain (Figure 1B), along with increased diarrhea rate (Figure 1C), elevated fecal water content (Figure 1D), bristol score (Figure 1E), and enhanced visceral sensitivity (Figure 1F), confirming successful model induction. After treatment with different doses of JFZX for 14 consecutive days, the reduction in body weight gain was significantly attenuated (Figure 1B). Moreover, JFZX administration markedly lowered the diarrhea rate and fecal water content, and bristol score (Figure 1C‐E) and alleviated visceral hypersensitivity (Figure 1F). Together, these data indicate that JFZX effectively improves key symptomatic features of IBS‐D.

**Figure 1:**
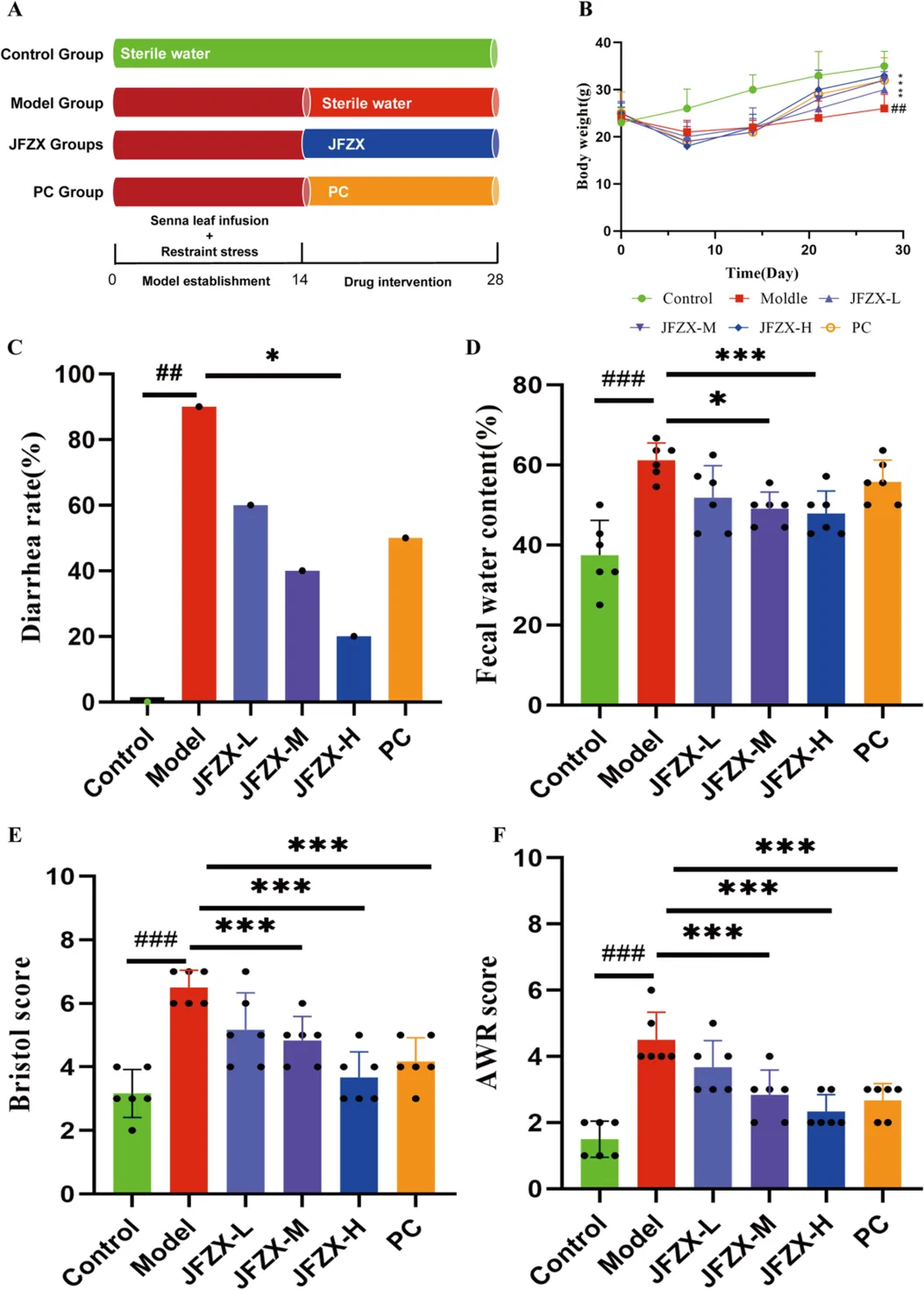
JFZX improves overall health and alleviates diarrheal symptoms in mice with IBS‐D. (A) Schematic representation of the establishment of the mouse model. (B) Changes in body weight. (C) Incidence of diarrhea across all groups. (D) Content of water in feces. (E) Bristol stool scale score. (F) AWR scoring. *N* = 10 for each group. ^#^ *p* < 0.05, ^##^ *p* < 0.01, ^###^ *p* < 0.001 compared to the control group; *p < 0.05, ***p < 0.001 compared to the Model group.

### 3.2 JFZX Ameliorated Intestinal Barrier Function in IBS‐D Mice

Intestinal morphology was evaluated by H&E and WB analysis. H&E staining demonstrated structural damage in the colonic mucosa of model mice, featuring the infiltration of inflammatory cells between the glands and a disordered tissue architecture. JFZX treatment alleviated these pathological changes and reduced inflammatory cell infiltration in a dose‐dependent manner (Figure 2A). Western blot analysis further demonstrated that JFZX administration upregulated the expression of the tight‐junction proteins ZO‐1 and Occludin in colonic tissues (Figure 2B).

**Figure 2:**
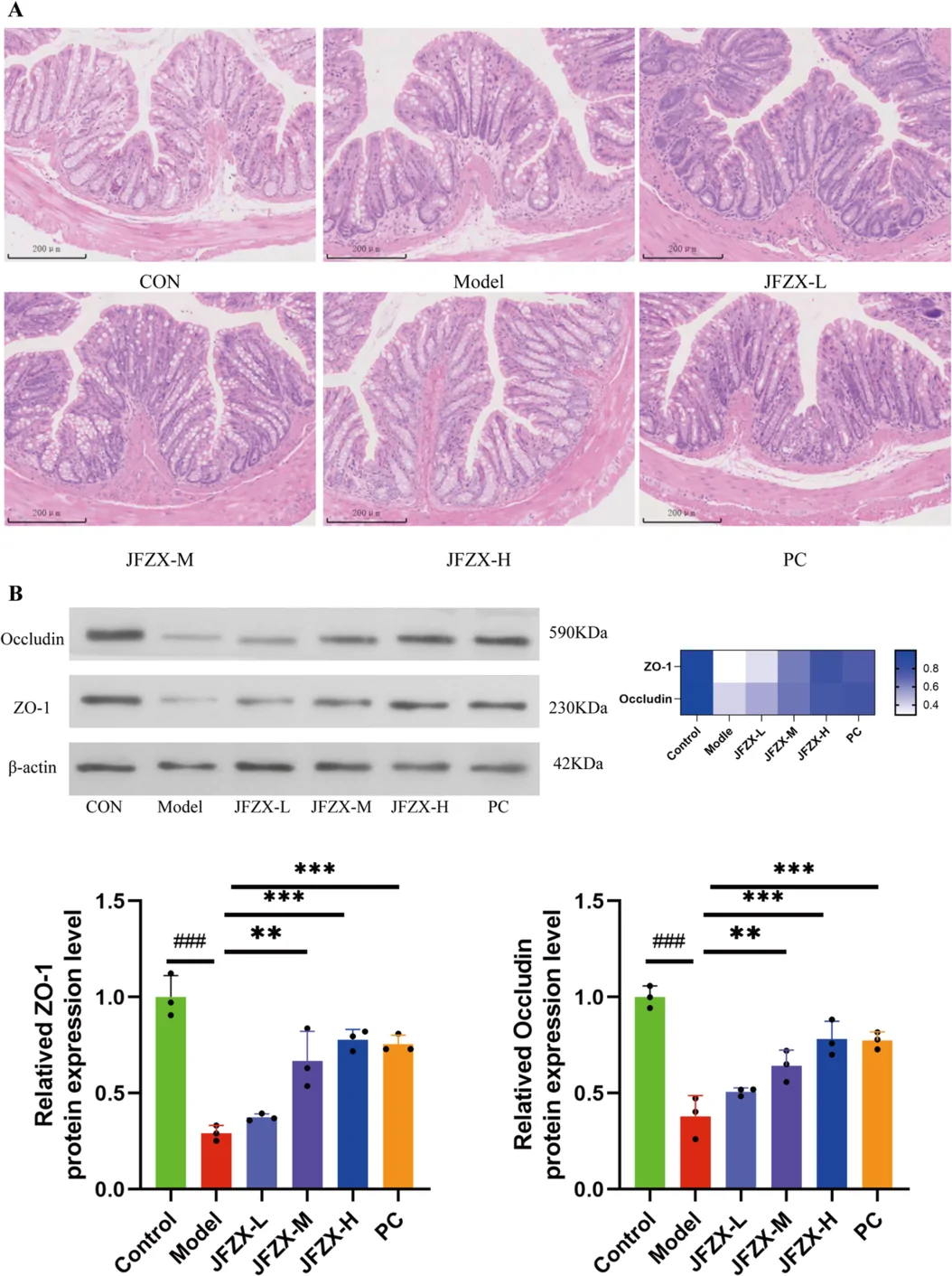
Impact of JFZX on histopathological changes and the functionality of the intestinal barrier. (A) Representative images of H&E staining from sections of intestinal tissue (scale bar: 100μm). (B) Results from Western blot analysis indicating the expression levels of ZO‐1 and occludin. Protein amounts were adjusted relative to β‐actin. *N* = 3 for each group, ^###^ *p* < 0.001 compared to the control group; **p < 0.01, ***p < 0.001 compared to the Model group.

### 3.3 scRNA‐Seq Reveals Altered Colonic Cellular Heterogeneity in IBS‐D Mice

To investigate the effect of JFZX on the colonic cellular landscape, we performed scRNA‐seq on colon tissues from Control, Model, and JFZX‐M treated mice (Figure 3A). After quality control and filtering, 18,847, 25,279, and 23,284 high‐quality cells from the Control, Model, and JFZX groups, respectively, were retained for analysis. Unsupervised clustering based on UMAP identified 22 distinct cell clusters (Figure 3B). Using established marker genes [24, 25], The analysis identified five major cell lineages within the specified clusters. These lineages include epithelial cells, characterized by the expression of markers such as Epcam and Krt8; stromal cells, which express *Col1a1*, *Col1a2*, *Col6a1*, and *Col6a2*; T cells, marked by the presence of *Cd3d*, *Cd3e*, and *Cd3g*; B cells, distinguished by the expression of *Cd79a* and *Cd79b*; and myeloid cells, encompassing *Cd14*, *Cd68*, and *Csf1r*. This categorization is illustrated in Figure 3C,D, providing a clear visualization of the distinct cell populations identified in the study. (Figure 3C,D).

**Figure 3:**
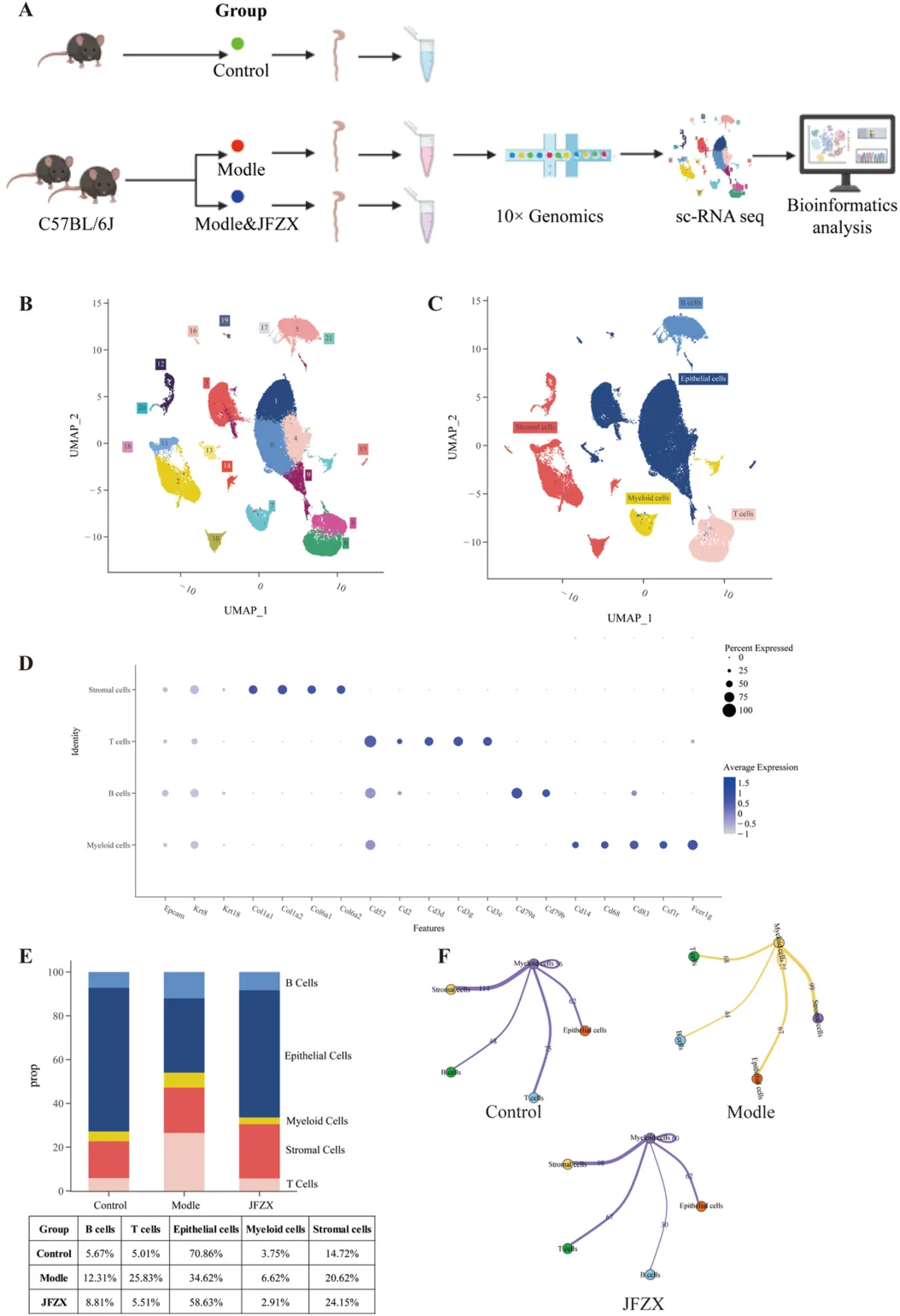
scRNA‐seq reveals JFZX‐induced alterations in colonic cellular heterogeneity in IBS‐D mice. (A) Schematic overview of the single‐cell RNA‐seq experimental workflow (created with BioRender). UMAP projections showing 22 minor clusters (B) and 5 major cell types (C).(D) Scatter plots depicting representative marker genes for the five major cell types.(E) Stacked bar charts showing the relative proportions of major cell types in the Control, Model, and JFZX groups.(F) Interaction strength between myeloid cells and other cell types in the Control, Model, and JFZX groups.

Comparative analysis of cellular composition revealed that JFZX treatment increased the proportions of epithelial cells (from 34.62% to 58.63%) and stromal cells (from 20.62% to 24.15%), while decreasing the proportions of B cells (12.81% to 8.81%), T cells (25.83% to 5.51%), and myeloid cells (6.62% to 2.91%) (Figure 3E). To assess changes in intercellular communication, we analyzed ligand–receptor interactions across groups. Compared with controls, model mice exhibited increased interaction number and strength between myeloid and epithelial cells. Conversely, JFZX treatment markedly attenuated these myeloid–epithelial interactions (Figure 3F), suggesting a role for myeloid cells in modulating epithelial homeostasis in the inflamed colon.

### 3.4 Association of M1‐like Macrophages With IBS‐D

Based on the alterations noted in the myeloid compartment after JFZX treatment, unsupervised sub‐clustering analysis was conducted on myeloid cells to explore their diversity [20, 26], five distinct myeloid subpopulations were identified (Figure 4A, B). Tissue‐resident‐like macrophages were characterized by elevated expression of *F13a1* and *Lyve1*, canonical markers for this population [27]. Monocytes, exhibiting high expression of *Ly6c2* and *Ccr2*, shared features with classical monocyte markers [28]. Macrophages that present antigens showed increased expression levels of H2‐Ab1, H2‐Aa, and H2‐Eb1, which are key genes associated with antigen presentation [29]. Macrophages resembling M1 exhibited an increased expression of genes like *Nlrp3*, *Il1b*, and *Cd86*, which are recognized as indicators of pro‐inflammatory pathological conditions [30].

**Figure 4:**
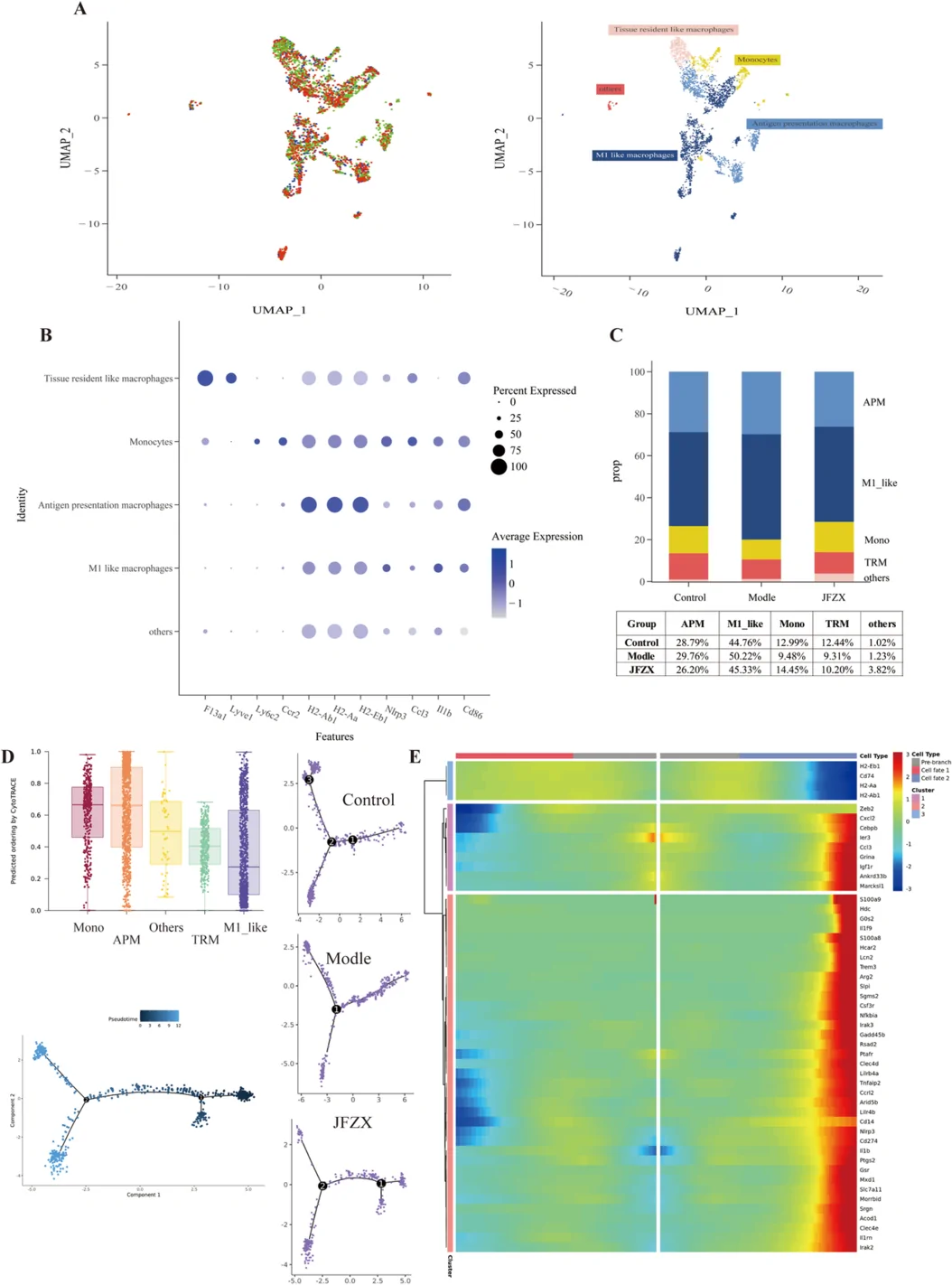
JFZX reshapes the composition of myeloid cells. (A) UMAP visualization showing the five major myeloid cell clusters (right) in the Control, Model, and JFZX groups (left, color‐coded: Control, green; Model, red; JFZX, blue). (B) Representative marker genes expressed in myeloid cell subsets. (C) Quantification of myeloid cell subset numbers in the Control, Model, and JFZX groups. APM, antigen‐presenting macrophages; M1_like, M1‐like macrophages; Mono, monocytes; TRM, tissue‐resident‐like macrophages. (D) Pseudotime trajectory analysis of myeloid cell differentiation. (E) Heatmap depicting the expression dynamics of selected genes along the pseudotime axis during myeloid cell fate bifurcation.

Subsequently, we analyzed the variations in the relative ratios of these five subpopulations after JFZX treatment. Importantly, there was an increase in the proportions of antigen‐presenting macrophages and M1‐like macrophages in the group that received JFZX treatment. Concurrently, an increase in Monocytes was also observed (Figure 4C).

To explore potential relationships among the myeloid subpopulations, we applied pseudotime analysis to study the dynamic immune states and transition processes. The analysis using CytoTRACE along with trajectory inference from Monocle2 revealed that Monocytes occupied the initial position on the pseudotime trajectory, whereas Antigen‐presenting macrophages, M1‐like macrophages, and Tissue‐resident‐like macrophages were situated closer to the endpoint (Figure 4D). By tracing gene expression changes along the monocyte‐to‐macrophage continuum, differentiation trajectories defining the functional characteristics of macrophage subsets were delineated. Based on the reconstructed pseudotime trajectory, we tracked genes significantly associated with the branching decision points. The branch leading to Antigen‐presenting macrophages exclusively expressed antigen presentation genes (H2‐Ab1, H2‐Eb1, H2‐Aa). The branch leading to M1‐like macrophages initiated expression of classical genes associated with macrophage polarization and pro‐inflammatory responses, such as *Nlrp3* and *Il1b* (Figure 4E).

### 3.5 Identification of Signaling Pathways and Regulatory Factors Specific to M1‐like Macrophages

To clarify the functional status of M1‑like macrophages in IBS‑D, we conducted a KEGG enrichment analysis on the genes that were expressed differently. The results showed significant enrichment in inflammatory pathways, such as chemokine signaling, NOD‑like receptor signaling, TNF signaling, and NF‑κB signaling (Figure 5A).

**Figure 5:**
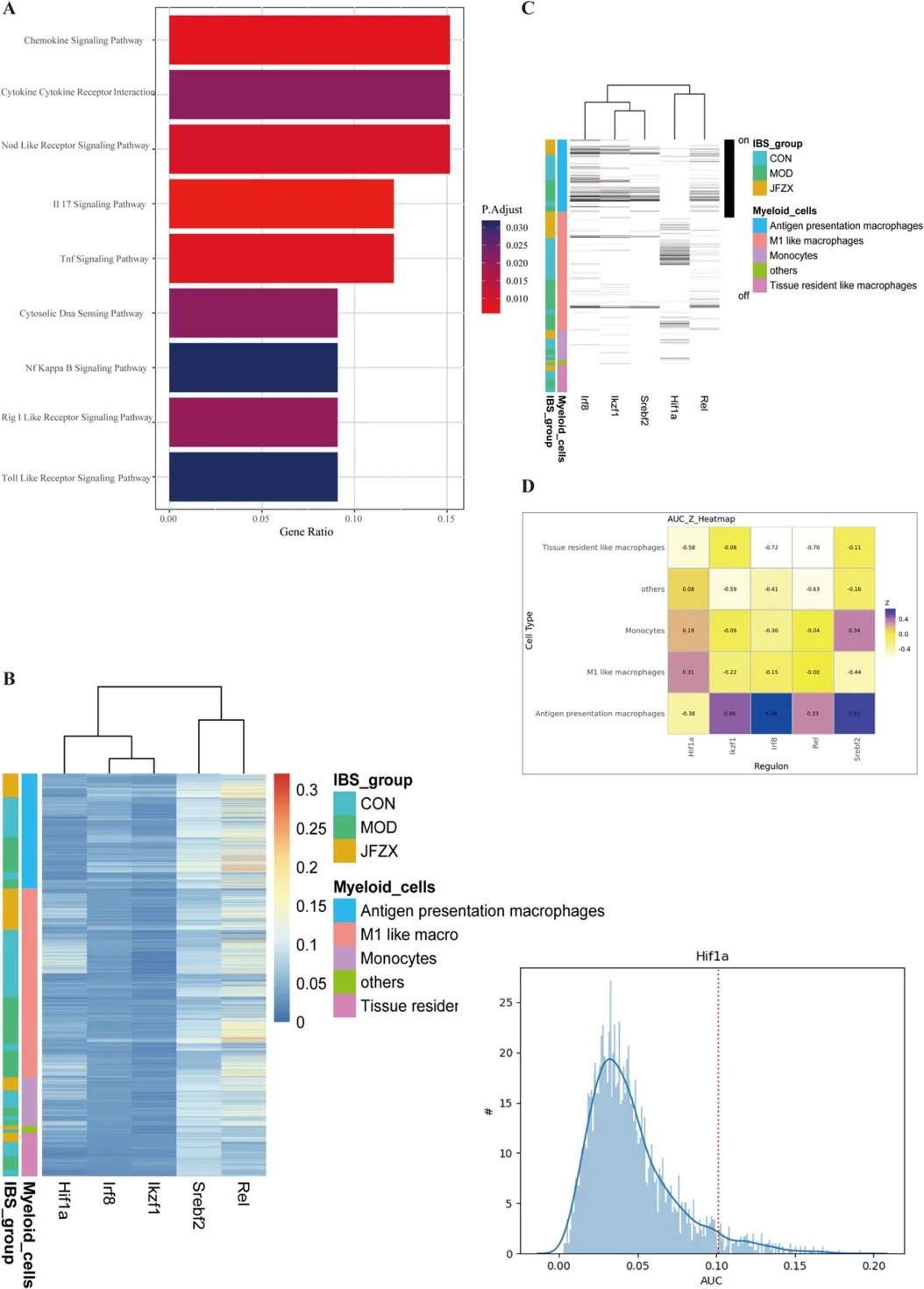
Different regulators for identity maintenance in macrophage subgroups. (A) KEGG enrichment analysis of genes upregulated in M1‐like macrophages. (B) GSEA revealing significant enrichment of the HIF‐1α signature between Model and JFZX groups. (C) Regulon activity heatmap. Rows represent regulons; columns represent individual cells. Black denotes an “ON” state (regulon active and accessible), white denotes an “OFF” state (regulon inactive). (D) HIF‐1α transcription factor expression across distinct cell types.

Given the established role of HIF‑1α as a molecular hub connecting glycolysis and macrophage polarization, we examined its activity in this context. Under hypoxic or inflammatory conditions, HIF‑1α is stabilized and drives transcriptional programs that promote both glycolytic metabolism and pro‑inflammatory (M1) polarization [31, 32]. Consistent with this, AUCell‑based regulator activity analysis revealed that HIF‑1α activity was markedly associated with M1‑like macrophages in IBS‑D (Figure 5B).

Further analysis of transcription‑factor regulon activity highlighted HIF‑1α as a key regulator. The number of cells with active HIF‑1α regulons was increased in IBS‑D, and regulon activity scoring confirmed that the HIF‑1α regulon was most active specifically in M1‑like macrophages (Figure 5C,D). These results position HIF‑1α as a central transcriptional driver of the pro‑inflammatory macrophage state in IBS‑D.

### 3.6 JFZX Inhibits LPS‐Induced M1 Macrophage Polarization In Vitro

To determine whether JFZX modulates macrophage polarization and inflammatory cytokine release, RAW 264.7 cells were stimulated with LPS (100 ng/mL) for 24 h to induce M1 polarization. CCK‐8 assays confirmed that JFZX‐medicated serum (2.5%–60%) did not significantly affect the viability of either naïve or LPS‐treated RAW 264.7 cells (Figure 6A,B). Accordingly, 10% JFZX‐medicated serum and a 48‐h incubation period were selected for all subsequent in vitro experiments. LPS stimulation markedly upregulated the M1‐associated marker CD86 and downregulated the M2‐associated marker CD206; both effects were substantially reversed by JFZX treatment (Figure 6C).

**Figure 6:**
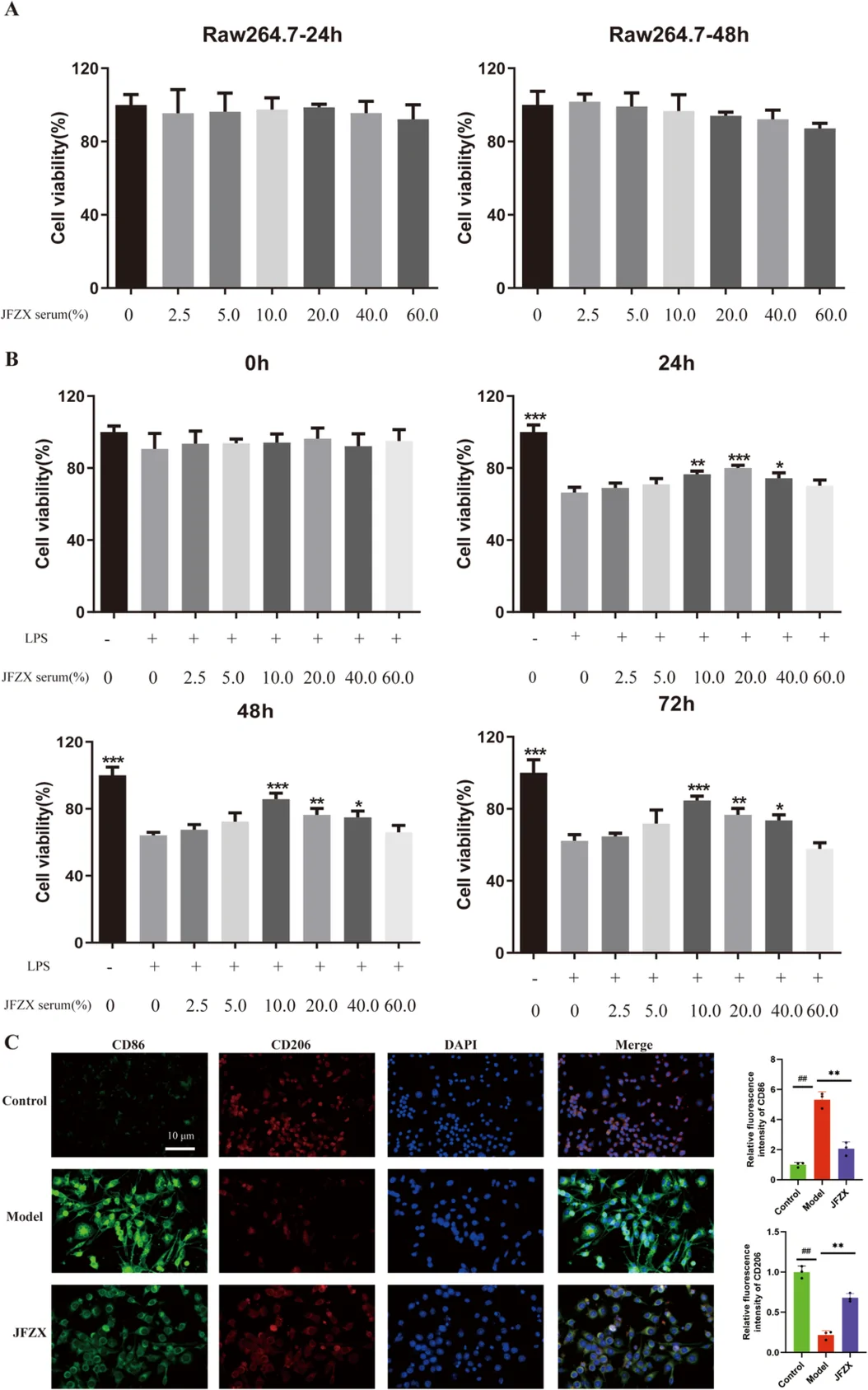
JFZX inhibits M1‐like macrophage polarization. The viability of Raw264.7 cells (A) and RAW 264.7 cells stimulated with LPS (B) was assessed after treatment with various concentrations of JFZX medicated serum. (C) The expression of CD86 and CD206 was detected using immunofluorescence imaging. *N* = 3 for each group. In (B), *p < 0.05, **p < 0.01, ***p < 0.001 compared to the LPS (+) and JFZX serum (‐) group. In (C), ^##^ *p* < 0.01 in relation to the Control group; ^###^ *p* < 0.001 compared to the Control group; **p < 0.01 in comparison to the Model group.

JFZX also significantly suppressed LPS‐induced expression of the pro‐inflammatory cytokines IL‐1β and TNF‐α (Figure 7A). WB analysis revealed that HIF‐1α protein levels were reduced in both intestinal tissues and cultured cells following JFZX treatment. Furthermore, JFZX treatment decreased the protein levels of GLUT1, a key glucose transporter, and LDHA, a rate‐limiting glycolytic enzyme (Figure 7B,C).

**Figure 7:**
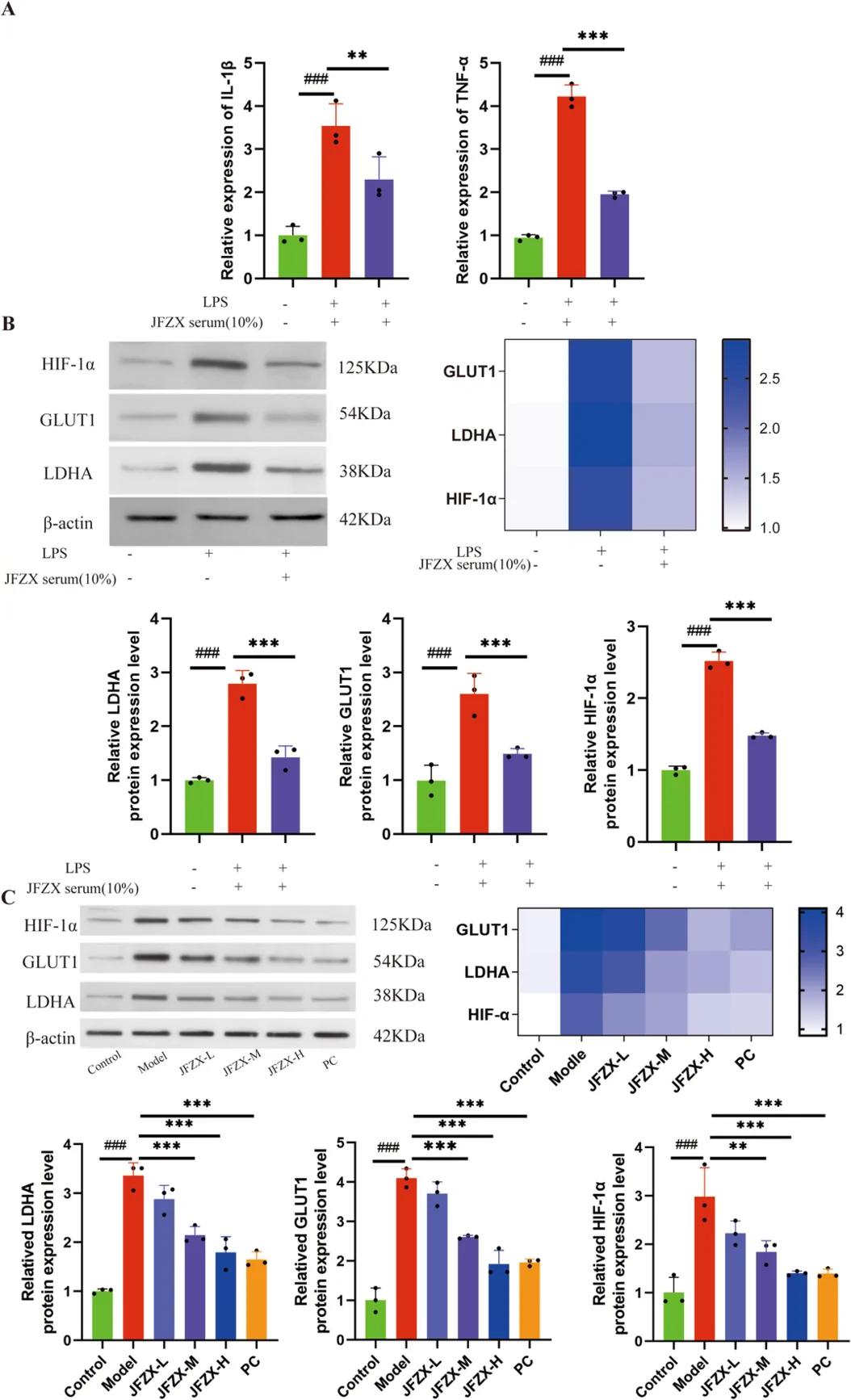
JFZX suppresses M1 macrophage polarization in a HIF‐1α/G‐LUT1/LDHA‐dependent manner. (A) Expression levels of IL‐1β and TNF‐α. Protein levels of HIF‐1α, GLUT1, and LDHA in RAW 264.7 cells (B) and col‐onic tissues (C) Protein expression levels were normalized against β‐actin. *N* = 3 for each group, with statistically significant differences indicated as ^###^ *p* < 0.001 compared to the Control group; **p < 0.01, and ***p < 0.001 compared to the Model group.

## 4 Discussion

IBS‐D is a common gastrointestinal condition, yet its exact pathophysiological mechanisms are not fully clarified. The prevailing pharmacological treatment primarily focuses on managing symptoms but is often constrained by issues such as toxicity, side effects, and the risk of developing drug resistance [1]. Therefore, there is a critical need to create new therapeutic options that offer better efficacy and safety for individuals suffering from IBS‐D.

Within the framework of Traditional Chinese Medicine (TCM), IBS‐D is not diagnosed as a distinct entity but is understood through its primary symptoms, chiefly ‘diarrhea’ (Xie Xie), and often associated with ‘abdominal pain’ (Fu Tong) or ‘depression syndrome’ (Yu Zheng) [19, 33, 34]. Against this background, Chinese herbal formulas like JFZX have gained attention for managing complex conditions such as IBS‐D. JFZX is structured around the TCM pattern commonly identified in IBS‐D: spleen‐kidney yang deficiency with cold‐dampness obstruction. The formula uses *Zingiber officinale Roscoe* (Gan Jiang) and *Aconitum carmichaelii Debx* (Fu Zi) as monarch herbs—their pungent, hot nature aims to warm yang and dispel cold. These are supported by *Atractylodes lancea (Thunb.) DC*. (Cang Zhu) and *Smilax glabra Roxb*. (Fu Ling) to strengthen the spleen and dry dampness. Minister herbs, including *Ephedra sinica Stapf* (Ma Huang), *Neolitsea cassia (L.) Kosterm*. (Gui Zhi), and *Boerhavia diffusa L*. (Xi Xin), help disperse cold and regulate water passages. Several assistant herbs balance the formula: *Paeonia lactiflora Pall*. (Bai Shao) and *Schisandra chinensis (Turcz.) Baill*. (Wu Wei Zi) regulate the liver and consolidate fluids; *Pueraria montana var. lobata (Willd.) Maesen & S.M. Almeida ex Sanjappa & Predeep* (Ge Gen) promotes fluid production; *Ziziphus jujuba Mill*. (Da Zao) tonifies the middle *jiao*; *Citrus reticulata Blanco* (Chen Pi) moves qi; and fresh *Zingiber officinale Roscoe* (Sheng Jiang) aids dispersion. *Glycyrrhiza glabra L*. (Gan Cao) serves as the guide herb, harmonizing the entire prescription to achieve a balanced effect that warms without overdrying, and disperses while containing. Overall, JFZX's design follows the classic TCM strategy of warming yang and resolving dampness, employing acrid herbs to disperse, sweet herbs to moderate, and sour herbs to astringe.

IBS‐D is closely associated with impaired intestinal barrier function, characterized by disruption of epithelial tight junctions [35, 36]. In our research, we observed a decrease in the expression of tight junction proteins ZO‐1 and Occludin in the colons of IBS‐D mice. Treatment with JFZX successfully increased their expression, suggesting that JFZX may help to improve damage to the intestinal epithelial barrier. Additionally, low‐grade mucosal inflammation and immune activation are acknowledged characteristics of IBS‐D [37, 38]. Intestinal macrophages, which are abundant in the gut mucosa, play pivotal roles in maintaining microbial homeostasis and regulating epithelial turnover [36, 39]. The functional plasticity of colonic macrophages allows them to adapt to the inflammatory microenvironment in IBS‐D, contributing to the disease's heterogeneity [40, 41]. Notably, M1‐polarized macrophages can initiate and sustain inflammation through the production of pro‐inflammatory cytokines [42]. In our IBS‐D model, colonic tissues showed marked pathological changes and inflammatory infiltration, accompanied by a shift toward M1 macrophage polarization. JFZX administration reversed this shift by reducing the proportion of M1‐like macrophages, suggesting that the therapeutic benefit of JFZX in IBS‐D may be mediated, at least in part, through modulation of intestinal macrophage polarization.

Recent advances in scRNA‐seq have enabled high‐resolution dissection of cellular heterogeneity, driving breakthroughs in identifying key cell subsets and molecular events in disease progression [43]. However, the application of scRNA‐seq to IBS‐D remains limited. Given the pronounced cellular diversity of the colon and the close relationship between altered molecular signatures and IBS‐D pathogenesis, a deeper understanding of colonic cellular heterogeneity at single‐cell resolution is essential for advancing prevention and treatment strategies. In this study, scRNA‐seq analysis of the IBS‐D mouse model revealed that JFZX reshaped the colonic cellular landscape. We identified pronounced interactions between myeloid and epithelial cells in IBS‐D, underscoring the central role of myeloid populations in disease pathophysiology. Further analysis demonstrated an elevated abundance of pro‐inflammatory M1‐like macrophages in the model, which was effectively reversed by JFZX treatment. KEGG enrichment analysis indicated that M1‐like macrophages were associated with activation of the NOD‐like receptor and TNF signaling pathways.

Hypoxia‐inducible factor‐1α (HIF‐1α) serves as a master transcriptional regulator of cellular adaptation to hypoxia and inflammation. Under normoxic conditions, HIF‐1α is hydroxylated and degraded via the VHL‐dependent ubiquitin pathway [44, 45]. In our study, HIF‐1α activity was notably elevated in M1‐like macrophages. Using an LPS‐stimulated RAW264.7 macrophage model, we further confirmed that JFZX suppresses M1 polarization by inhibiting the HIF‐1α‐mediated glycolytic pathway, thereby reducing the production of pro‐inflammatory cytokines.

In conclusion, this study provides evidence that JFZX alleviates key symptoms and intestinal pathology in a murine model of IBS‑D, primarily by modulating macrophage polarization through inhibition of the HIF‑1α‑glycolysis axis. These findings offer a novel molecular perspective on the therapeutic action of JFZX and highlight HIF‑1α as a potential target for IBS‑D management.

Several limitations of this study should be acknowledged. First, as a multi‑herb formulation, JFZX contains numerous bioactive constituents, and the specific compounds responsible for the observed therapeutic effects remain to be fully elucidated. Future studies integrating metabolomics profiling with network pharmacology are warranted to systematically characterize the active components of JFZX and clarify its pharmacodynamic material basis. Second, the IBS‐D mouse model employed in this study was established by senna leaf infusion combined with restraint stress, which, while widely used and validated, may not fully recapitulate the complexity and heterogeneity of human IBS‐D. Third, although scRNA‐seq provided high‐resolution insights into cellular heterogeneity and cell‑cell interactions, it lacks spatial context. Complementary spatial transcriptomics approaches would be valuable to map these cellular changes and interactions within their native tissue architecture, thereby providing a more comprehensive understanding of the colon microenvironment in IBS‐D. Fourth, the in vitro experiments were performed using a murine macrophage cell line; primary human intestinal macrophages or patient‑derived samples would strengthen the translational relevance of our findings. Addressing these points in subsequent investigations will further solidify the mechanistic understanding and enhance the translational potential of JFZX for IBS‐D management.

## 5 Conclusion

In summary, our study demonstrates that JFZX alleviates key symptoms of IBS‐D—including diarrhea and visceral hypersensitivity—and enhances intestinal barrier integrity in a murine model. Through single‐cell transcriptomic analysis, we delineated the colonic cellular landscape in IBS‐D and identified a critical role for pro‐inflammatory M1‐like macrophages. Mechanistically, we identified HIF‐1α as a central transcriptional driver of M1 polarization, and confirmed that JFZX inhibits HIF‐1α‐mediated glycolysis, thereby suppressing macrophage M1 polarization and downstream inflammatory cytokine release in vitro. These findings collectively indicate that the therapeutic effect of JFZX against IBS‐D is mediated, at least in part, through modulation of the HIF‐1α‐glycolysis axis and subsequent restraint of pathogenic macrophage polarization (Figure 8). To further advance this work, future studies should focus on: 1) elucidating the bioactive constituents of JFZX responsible for modulating macrophage polarization; 2) identifying biomarkers linked to macrophage phenotypic states; and 3) dissecting the spatial and functional crosstalk between macrophages and epithelial cells in the IBS‐D microenvironment.

**Figure 8:**
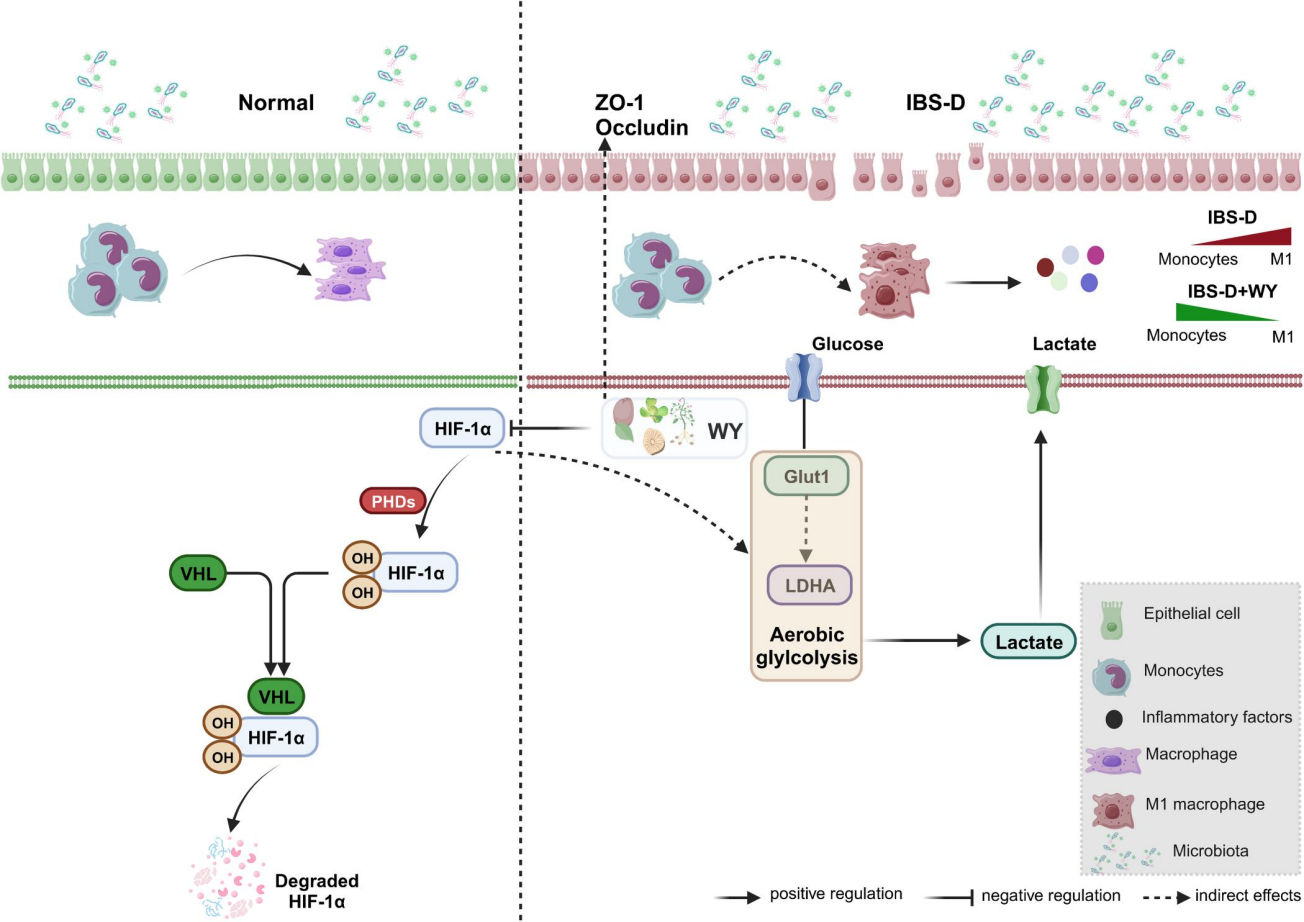
Mechanism diagram of JFZX. (created with CNSknowall).

## Author Contributions

**Xiaosong Xu:** Methodology, Writing – original draft, Writing – review and editing. **Yao Du:** Methodology, Writing – original draft, Writing – review and editing. **Lipeng Du:** Methodology. **Wanning Zhang:** Writing – original draft. **Bowen Guo:** Writing – review and editing. **Yanru Cai:** Resources. **Tianyi Zhang:** Validation.

## Ethics Statement

The protocol for animal study received approval from the Ethics Committee on Animal Experimentation and Welfare at Hebei Provincial Hospital of Traditional Chinese Medicine. All experimental methods were executed in full compliance with the applicable ethical standards and regulations.

## Conflicts of Interest

The authors declare no conflicts of interest.

## Declaration of Generative AI

During the preparation of this manuscript, the authors did not use any generative AI or AI‐assisted technologies for the writing, data analysis, or interpretation of findings. All content, including the experimental design, data interpretation, and manuscript drafting, was performed by the authors independently. The authors take full responsibility for the integrity and accuracy of the content presented in this paper.

## Supporting information


**Table S1:** Detailed information of herbs in JFZX.

**Table S2:** Abdominal Withdrawal Reflex (AWR) Scoring.

Supporting File

## Data Availability

The data that support the findings of this study are available on request from the corresponding author. The data are not publicly available due to privacy or ethical restrictions.
