# Supplementary material for: ScRNA‐Seq Reveals Jiangfu Zhixie Formula Alleviates IBS‐D by Inhibiting Macrophage M1 Polarization through HIF‐1α‐Glycolysis Axis
Source: Immun Inflamm Dis. 2026 Nov 5;14(11):e70599. doi: 10.1002/iid3.70599 (PMC13737473; doi:10.1002/iid3.70599)
Supplement: Supplementary file 1 [file IID3-14-e70599-s001.pdf]

NOTE: Please save this file locally before filling in the table, DO NOT work on the file within your internet browser as changes will not be saved. Adobe Acrobat Reader (available free here) is recommended for completion.

# The ARRIVE guidelines 2.0: author checklist

## The ARRIVE Essential 10

These items are the basic minimum to include in a manuscript. Without this information, readers and reviewers cannot assess the reliability of the findings.

| Item | | Recommendation | Section/line number, or reason for not reporting |
|---|---|---|---|
| **Study design** | 1 | For each experiment, provide brief details of study design including: | |
| | | a. The groups being compared, including control groups. If no control group has been used, the rationale should be stated. | Control, Model, JFZX-L, JFZX-M, JFZX-H, PC (pinaverium bromide). |
| | | b. The experimental unit (e.g. a single animal, litter, or cage of animals). | Single mouse. |
| **Sample size** | 2 | a. Specify the exact number of experimental units allocated to each group, and the total number in each experiment. Also indicate the total number of animals used. | n=10/group, 6 groups, total 60 mice. |
| | | b. Explain how the sample size was decided. Provide details of any *a priori* sample size calculation, if done. | Based on previous studies and institutional guidelines; no formal power calculation performed. |
| **Inclusion and exclusion criteria** | 3 | a. Describe any criteria used for including and excluding animals (or experimental units) during the experiment, and data points during the analysis. Specify if these criteria were established *a priori*. If no criteria were set, state this explicitly. | No inclusion/exclusion criteria were set a priori; all animals completed the study. |
| | | b. For each experimental group, report any animals, experimental units or data points not included in the analysis and explain why. If there were no exclusions, state so. | No exclusions; all 60 mice and data points were included. |
| | | c. For each analysis, report the exact value of *n* in each experimental group. | Animal studies: n=10/group. Cell assays: n=3 replicates/group. |
| **Randomisation** | 4 | a. State whether randomisation was used to allocate experimental units to control and treatment groups. If done, provide the method used to generate the randomisation sequence. | Yes, random division |
| | | b. Describe the strategy used to minimise potential confounders such as the order of treatments and measurements, or animal/cage location. If confounders were not controlled, state this explicitly. | Randomization only; no additional measures for order or cage location. |
| **Blinding** | 5 | Describe who was aware of the group allocation at the different stages of the experiment (during the allocation, the conduct of the experiment, the outcome assessment, and the data analysis). | Not blinded; group allocation known to investigators throughout. |
| **Outcome measures** | 6 | a. Clearly define all outcome measures assessed (e.g. cell death, molecular markers, or behavioural changes). | tol score, AWR score, body weight, H&E histology, tight-junction proteins (ZO-1, Occludin) by WB, cell composition/interactions by scRNA-seq, CD86/CD206 by IF, IL-1β/TNF-α by ELISA, HIF-1α/GLUT1/LDHA by WB. |
| | | b. For hypothesis-testing studies, specify the primary outcome measure, i.e. the outcome measure that was used to determine the sample size. | No formal power calculation; sample size based on prior literature, so no primary outcome specified for sample size determination. |
| **Statistical methods** | 7 | a. Provide details of the statistical methods used for each analysis, including software used. | GraphPad Prism 8.0; one-way ANOVA with Dunnett's post hoc; data as mean±SD; significance at P<0.05. |
| | | b. Describe any methods used to assess whether the data met the assumptions of the statistical approach, and what was done if the assumptions were not met. | re assumed based on equal group sizes (n=10) and prior literature. If violated, non?parametric tests (Kruskal?Wallis) or data transformation would be used, but no violations were observed, so no adjustments were applied. |
| **Experimental animals** | 8 | a. Provide species-appropriate details of the animals used, including species, strain and substrain, sex, age or developmental stage, and, if relevant, weight. | Male C57BL/6 mice, 8 weeks old, 18–20 g, SPF. |
| | | b. Provide further relevant information on the provenance of animals, health/immune status, genetic modification status, genotype, and any previous procedures. | Beijing HFK Bioscience, SPF, wild-type, no prior procedures. |
| **Experimental procedures** | 9 | For each experimental group, including controls, describe the procedures in enough detail to allow others to replicate them, including: | |
| | | a. What was done, how it was done and what was used. | mg/kg/day; all oral gavage, daily for 14 days. Colon harvested after overnight fast under pentobarbital anaesthesia; processed for histology, WB, scRNA?seq, etc. |
| | | b. When and how often. | Model induction: daily for 14 days; treatment: daily for 14 days; sampling: after overnight fast, on day 15. |
| | | c. Where (including detail of any acclimatisation periods). | SPF facility, Hebei Provincial Hospital of Chinese Medicine; 1-week acclimation. |
| | | d. Why (provide rationale for procedures). | nsitivity per published protocol. JFZX dose based on body surface conversion from human use. Pinaverium bromide as standard positive control. 14?day treatment based on preliminary efficacy data. |
| **Results** | 10 | For each experiment conducted, including independent replications, report: | |
| | | a. Summary/descriptive statistics for each experimental group, with a measure of variability where applicable (e.g. mean and SD, or median and range). | Data: mean±SD; animal studies n=10/group, cell assays n=3/group; all summary values (weight, diarrhea, fecal water, Bristol, AWR, protein, cytokines) shown in figures with error bars and legends. |
| | | b. If applicable, the effect size with a confidence interval. | Not applicable; effect sizes not reported. |

# The Recommended Set

These items complement the Essential 10 and add important context to the study. Reporting the items in both sets represents best practice.

| Item | | Recommendation | Section/line number, or reason for not reporting |
|---|---|---|---|
| **Abstract** | 11 | Provide an accurate summary of the research objectives, animal species, strain and sex, key methods, principal findings, and study conclusions. | rization through HIF?1α?glycolysis axis. |
| **Background** | 12 | a. Include sufficient scientific background to understand the rationale and context for the study, and explain the experimental approach. | ?1α?glycolysis axis. We used senna + restraint stress IBS?D mouse model, JFZX treatment (low/mid/high) vs. control/PC, assessed symptoms, barrier, scRNA?seq, and in vitro LPS?stimulated RAW264.7 with JFZX serum to test the hypothesis. |
| | | b. Explain how the animal species and model used address the scientific objectives and, where appropriate, the relevance to human biology. | raint stress model recapitulates human IBS?D features (diarrhea, visceral hypersensitivity, barrier dysfunction) and allows mechanistic dissection of inflammation and immune regulation, providing translational relevance for human IBS?D therapy. |
| **Objectives** | 13 | Clearly describe the research question, research objectives and, where appropriate, specific hypotheses being tested. | mmation, and restores intestinal barrier. |
| **Ethical statement** | 14 | Provide the name of the ethical review committee or equivalent that has approved the use of animals in this study, and any relevant licence or protocol numbers (if applicable). If ethical approval was not sought or granted, provide a justification. | Ethics Committee on Animal Experimentation and Welfare, Hebei Provincial Hospital of TCM; approval No. IACUC-HPHCM-2024105. |
| **Housing and husbandry** | 15 | Provide details of housing and husbandry conditions, including any environmental enrichment. | environmental enrichment details provided. |
| **Animal care and monitoring** | 16 | a. Describe any interventions or steps taken in the experimental protocols to reduce pain, suffering and distress. | Pentobarbital anesthesia for tissue collection |
| | | b. Report any expected or unexpected adverse events. | No adverse events observed. |
| | | c. Describe the humane endpoints established for the study, the signs that were monitored and the frequency of monitoring. If the study did not have humane endpoints, state this. | e.g., severe weight loss, lethargy, abnormal behavior); none occurred. All procedures were approved by the ethics committee, and if severe suffering had been observed, affected animals would have been humanely euthanized. |
| **Interpretation/ scientific implications** | 17 | a. Interpret the results, taking into account the study objectives and hypotheses, current theory and other relevant studies in the literature. | macrophage metabolic reprogramming. scRNA-seq reveals JFZX remodels colonic cellular interactions, reducing myeloid-epithelial crosstalk. These findings support the hypothesis and identify HIF-1α as a promising therapeutic target. |
| | | b. Comment on the study limitations including potential sources of bias, limitations of the animal model, and imprecision associated with the results. | human IBS-D; ②No formal sample size calculation; ③No blinding, potential bias; ④In vitro only murine cell line, human validation needed; ⑤scRNA-seq lacks spatial context; ⑥Bioactive components of JFZX not identified. |
| **Generalisability/ translation** | 18 | Comment on whether, and how, the findings of this study are likely to generalise to other species or experimental conditions, including any relevance to human biology (where appropriate). | The findings may be partially generalizable to other mammals due to conserved HIF-1α–glycolysis pathways, but direct human translation requires caution: the IBS-D model does not fully capture human heterogeneity, and RAW264.7 cells differ from primary human macrophages. Human validation and clinical studies are needed. |
| **Protocol registration** | 19 | Provide a statement indicating whether a protocol (including the research question, key design features, and analysis plan) was prepared before the study, and if and where this protocol was registered. | No formal protocol was pre?registered; the study design and analysis plan were not registered in any public repository. |
| **Data access** | 20 | Provide a statement describing if and where study data are available. | ly available due to ethical restrictions. |
| **Declaration of interests** | 21 | a. Declare any potential conflicts of interest, including financial and non-financial. If none exist, this should be stated. | No conflicts of interest declared. |
| | | b. List all funding sources (including grant identifier) and the role of the funder(s) in the design, analysis and reporting of the study. | of Hebei Administration of TCM (2022032, 2024010, 2026240); Medical Science Research Project of Hebei (20250859). Role of funders: None; they had no role in study design, data collection, analysis, or manuscript preparation. |
