# Supplementary material for: ScRNA‐Seq Reveals Jiangfu Zhixie Formula Alleviates IBS‐D by Inhibiting Macrophage M1 Polarization through HIF‐1α‐Glycolysis Axis
Source: Immun Inflamm Dis. 2026 Nov 5;14(11):e70599. doi: 10.1002/iid3.70599 (PMC13737473; doi:10.1002/iid3.70599)
Supplement: Supplementary file 2 [file IID3-14-e70599-s002.docx]

Table S2 Abdominal Withdrawal Reflex (AWR) Scoring.

| Mice behavior altering | Score |
| --- | --- |
| Mouse displays emotional stability, no apparent discomfort behavior | 0 |
| Emotional instability, occasional head twisting movements | 1 |
| Mild contraction of abdominal and back muscles, but abdomen remains in contact with the ground | 2 |
| Moderate to strong contraction of abdominal and back muscles, lifting the abdomen off the ground | 3 |
| Strong contraction of abdominal muscles, forming an arched position, lifting the abdomen and perineal area off the ground | 4 |
