# Supplementary material for: ScRNA‐Seq Reveals Jiangfu Zhixie Formula Alleviates IBS‐D by Inhibiting Macrophage M1 Polarization through HIF‐1α‐Glycolysis Axis
Source: Immun Inflamm Dis. 2026 Nov 5;14(11):e70599. doi: 10.1002/iid3.70599 (PMC13737473; doi:10.1002/iid3.70599)
Supplement: Supplementary file 3 [file IID3-14-e70599-s003.docx]

Table S1 Detailed information of herbs in WY.

| Chinese name | **Latin name** | **Part(s) used** | **Amount (g)** |
| --- | --- | --- | --- |
| Fuzi | Aconitum carmichaelii Debx | Sub-root | 9 |
| Ganjiang | Zingiber officinale Roscoe | roots and rhizomesroot | 6 |
| Mahuang | Ephedra sinica Stapf | Stem | 6 |
| Guizhi | Neolitsea cassia (L.) Kosterm. | Branch | 6 |
| Xixin | Boerhavia diffusa L. | root and rhizomesroot | 3 |
| Baishao | Paeonia lactiflora Pall. | root | 15 |
| Wuweizi | Schisandra chinensis (Turcz.) Baill. | fruit | 6 |
| Chenpi | Citrus reticulata Blanco | rind | 10 |
| Fuling | Smilax glabra Roxb. | sclerotium | 15 |
| Cangzhu | Atractylodes lancea (Thunb.) DC. | root and rhizomesroot | 9 |
| Gegen | Pueraria montana var. lobata (Willd.) Maesen & S.M.Almeida ex Sanjappa & Predeep | root | 15 |
| Dazao | Ziziphus jujuba Mill. | fruits | 10 |
| Gancao | Glycyrrhiza glabra L. | root and rhizomesroot | 10 |
| Shengjiang | Zingiber officinale Roscoe | roots and rhizomesroot | 6 |
